# Restructuring of Femoral Cortical Bone During Growth and Locomotor Development of Wild Chimpanzees ( *Pan troglodytes verus* )

**DOI:** 10.1002/ajpa.25045

**Published:** 2024-11-30

**Authors:** Karen R. Swan, Zewdi J. Tsegai, Rachel Ives, Louise T. Humphrey

**Affiliations:** ^1^ Centre for Human Evolution Research (CHER) Natural History Museum London UK; ^2^ Department of Organismal Biology and Anatomy University of Chicago Chicago Illinois USA; ^3^ Vertebrates and Anthropology Collections Natural History Museum London UK

**Keywords:** bone modeling, cortical bone porosity, cross‐sectional geometry, great ape, ontogeny

## Abstract

**Objective:**

Chimpanzees are altricial in terms of their locomotor development and transition from being carried to engaging in suspensory and arboreal locomotor behaviors to eventually relying on terrestrial quadrupedalism as their main form of locomotion. Here, we consider the mechanical implications of femoral cortical bone restructuring during growth and locomotor development in wild chimpanzees.

**Materials and Methods:**

Cortical bone structure was examined in an ontogenetic sample of wild chimpanzees from a single subspecies (*P. t. verus*) spanning in age from 2 weeks to 12.6 years. Diaphyseal cross‐sections were extracted from micro‐CT scans of the femur at 35%, 50%, and 65% of total intermetaphyseal length and variation in cortical bone structure was assessed based on bending rigidity (Imax/Imin, Ix/Iy), relative medullary area, and cortical bone porosity.

**Results:**

Diaphyseal shape is relatively circular with a high amount of cortical bone porosity and a large relative medullary area during early infancy. Distinct shifts in cortical bone structure occurred for each studied parameter with the biggest changes occurring within the first 5 years. Values appear to stabilize as quadrupedal walking increases in frequency and is established as the main form of locomotion.

**Discussion:**

Collectively, the results suggest a degree of integration in which cortical bone restructures in response to rapid changes in locomotion in addition to nonmechanical influences such as hormonal, and growth factors, without compromising function and structural integrity. The extent of influence of each factor varies throughout growth and highlights the need for caution in functional interpretations of cortical bone geometry.

## 1 Introduction

The structural composition of long bone diaphyses undergoes dynamic changes throughout life as a result of modeling and remodeling processes occurring at the periosteal, endosteal, and intracortical bone surfaces (Parfitt 1994). The amount of bone and how it is distributed within a diaphyseal cross‐section influences its mechanical resistance to bending and breakage, and is influenced by mechanical, hormonal, and metabolic factors (Frost 1987; Seeman 2008; Swan, Humphrey, and Ives 2023). Several studies have documented marked ontogenetic changes in cortical bone structure of modern humans, which have been associated with postnatal shifts in hormones, rapid growth, and increasing body mass, along with changes in biomechanical loading relating to locomotor development (Cowgill et al. 2010; Swan, Humphrey, and Ives 2023; Swan et al. 2020; Welsh and Brickley 2022). As our closest living relative, chimpanzees are highly relevant for the reconstruction of locomotor behaviors and developmental processes of extinct hominins. Yet, with distinct differences in locomotor specialism and phylogeny, it is not fully understood whether chimpanzees undergo a similar pattern of development of cortical bone structure to humans and, if so, whether they closely follow human trajectories. This paper aims to document multiple aspects of cortical bone structure of the femur in an ontogenetic sample of wild chimpanzees from a single subspecies (*P. t. verus*) and considers the mechanical implications of these changes during periods of rapid increase in body mass and changes in locomotor and postural behaviors. Here, we seek to obtain a broader understanding of the impact of mechanical and nonmechanical influences on developing bone structure.

### 1.1 Chimpanzee Locomotor Development

**FIGURE 1:**
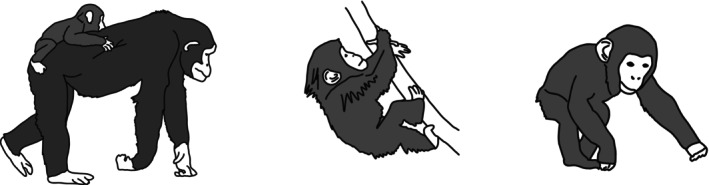
Illustrations of key stages of locomotor development in chimpanzees, which include from left to right; passive carrying and active clinging to mother, forelimb dominated climbing and suspensory behaviors and quadrupedal knuckle‐walking.

The primary locomotor mode of adult chimpanzees is knuckle‐walking quadrupedalism where fore‐and hindlimbs alternate using both lateral and diagonal sequence footfall patterns (Finestone et al. 2018; Pontzer, Raichlen, & Rodman, 2014) with higher peak vertical forces exerted on the hindlimbs (Demes et al. 1994). As chimpanzees maneuver in both terrestrial and arboreal environments, a range of other postural and locomotor behaviors, including climbing and occasional bipedalism, are also adopted at lesser and varying frequencies (Hunt 1992; Sarringhaus, MacLatchy, and Mitani 2014). However, like humans, chimpanzees are not born with the ability to locomote independently and undergo a series of locomotor transitions during the first few years of life (Figure 1). Newborn chimpanzees are initially unable to support their own body weight and for the first 2 months are carried by the mother (Doran 1992; Plooij 1984). At about 3 months, young infants are still carried but start to partially support their own body weight by actively clinging to the mother's fur in either ventral or dorsal positions (Plooij 1984). With decreasing dependency on the mother for locomotion, young chimpanzees gradually increase independent movements in their locomotor repertoire by primarily engaging in arboreal and suspensory behaviors and experimenting with bipedal and quadrupedal postures. This culminates with the adoption of terrestrial quadrupedal walking, which increases in predominance through to adulthood as body size increases and arboreal behaviors become less frequent (Doran 1992; Sarringhaus, MacLatchy, and Mitani 2014). There is some ambiguity in the perceived age of locomotor independence in chimpanzees, but it is generally considered to occur at approximately 5 years when infants are carried for a maximum of 20% of their daily transport (Doran 1992; Hayashi and Matsuzawa 2017; Sarringhaus, MacLatchy, and Mitani 2014; Young and Shapiro 2018). This broadly coincides with the approximate age of nutritional independence from the mother (Lonsdorf et al. 2020; Pusey 1983; Samuni et al. 2020).

### 1.2 Cross‐Sectional Geometry

The amount and distribution of cortical bone is partly influenced by mechanical loading via the process of bone functional adaptation where bone structure reflects the direction of loading, load frequency, and magnitude (Mosley and Lanyon 1998; Ruff, Holt, and Trinkaus 2006). The modeling of long bones as simple beams and the extraction of cross‐sectional properties has been utilized as a tool to approximate long bone strength and bending rigidity. Typically, diaphyseal cross‐sections that present an elliptical cross‐sectional shape are indicative of increased rigidity along specific axes in response to repeated unidirectional loading whereas more circular shapes are associated with minimal or multidirectional loading (Ruff and Hayes 1983). Interpretations of mechanical activity from cross‐sectional properties are complicated by potential impacts of nonmechanical factors and studies have challenged their correspondence with actual patterns of in vivo strains (Demes et al. 2001, 1998; Lieberman, Polk, and Demes 2004). While maintaining a degree of caution, previous research on primates has demonstrated broad differences in hindlimb to forelimb bending rigidity ratios, which appear to correspond with differences in locomotor strategies. For example, species with a low femoral‐humeral ratio tend to exhibit greater frequencies of forelimb dominated arboreal and suspensory behaviors in contrast to species with higher ratios, which are commonly quadrupedal (Ruff 2002; Sarringhaus et al. 2022; Schaffler et al. 1985). Several studies have demonstrated ontogenetic changes in primate diaphyseal shape that correspond with behavioral shifts during locomotor development, including the transition from crawling to walking and the development of a mature walking gait in humans (Cowgill and Johnston 2018; Cowgill et al. 2010; Ruff 2003; Swan et al. 2020). By comparing femoral cross‐sectional measurements with ground reaction force data from living children and adults, Cowgill et al. (2010) were able to present strong evidence for a close relationship between cross‐sectional geometry and the direction and frequency of bone loading. Early walkers aged under 4 years, were shown to experience higher mediolateral bending forces, which is reflected in a mediolateral elliptical shaped femoral diaphysis in comparison to mature walkers that have a diaphysis that is more reinforced in the anteroposterior direction. Previous research suggests similar developmental changes occur in chimpanzees as they develop their locomotor and postural repertoire where femoral/humeral strength and rigidity ratios increased with age reflecting an increasing mechanical demand placed on the femur as quadrupedal locomotor behaviors increase in frequency (Burgess 2018; Sarringhaus, MacLatchy, and Mitani 2014). Shape changes in the midshaft of the femur have been demonstrated to increase in mediolateral eccentricity during growth (Bleuze 2024; Burgess 2018; Sarringhaus, MacLatchy, and Mitani 2016). In comparison to humans and gorillas, these shape changes were observed to be more gradual which could reflect the variability of chimpanzee locomotion (Sarringhaus, MacLatchy, and Mitani 2016). More recently, Nadell, Elton, and Kovarovic (2021) measured cross‐sectional shape of the femur and humerus at two diaphyseal locations (midshaft, 50% and distal, 20%) in an ontogenetic sample of chimpanzees and offer an alternative explanation for changes in mid‐femoral shape. While humeral midshaft appears to correspond to changing locomotor patterns, the femoral midshaft more closely reflects shifts in body mass. Interestingly, the results also suggest differences in the pattern of shape changes relating to diaphyseal location. Shifts in cross‐sectional shape were detected at the mid‐section of both bones yet the distal location, at 20% of diaphyseal length, remained relatively static throughout the growth period suggesting shape constraints due to the requirement for stability at the knee joint (Nadell and Shaw 2016).

### 1.3 Relative Cortical Bone and Medullary Areas

The proportions of cortical bone and medullary cavity comprising the total subperiosteal cross‐section have been shown to undergo a marked shift during early postnatal growth in both human and non‐human primates (Cosnefroy et al. 2022; Swan et al. 2020). Previous research has demonstrated a relatively high proportion of cortical bone area (CA) at birth, which then decreases due to a rapid expansion of the medullary cavity. The combined effect of an increase in bone resorption at the endocortical margin and bone deposition at the periosteal surface is a redistribution of cortical bone further from the centroid of the cross‐section. This appears to stabilize as habitual limb loading commences. In humans, this stabilization corresponds to the time of mixed independent limb loading (e.g., unsupported sitting and crawling) from 6 months of age (Swan et al. 2020) and in olive baboons it occurs during a period characterized by the onset of independent locomotor behaviors (Cosnefroy et al. 2022). In both species, a rapid redistribution of cortical bone appears to occur prior to the age of complete locomotor independence. This postnatal redistribution of cortical bone is not a mechanically driven process but does provide a functional advantage by optimizing the distribution of bone mass while also acting as a calcium reservoir for the developing skeleton (Kontulainen et al. 2007; Rauch and Schoenau 2001; Swan et al. 2020).

A similar pattern of postnatal bone restructuring has been demonstrated in the trabecular bone microstructure of humans and Japanese macaques involving a high bone volume fraction (BV/TV) at birth which rapidly decreases in value until the onset of independent locomotor behaviors (Colombo et al. 2019; Milovanovic et al. 2017; Ryan and Krovitz 2006; Ryan, Raichlen, and Gosman 2017; Saers et al. 2022). Interestingly, this pattern is absent in the proximal humerus, distal tibia, and proximal femur of wild chimpanzees (Tsegai et al. 2018a). While acknowledging that small sample size may contribute to the absence of this pattern, Tsegai et al. (2018a) encouraged further investigation into the existence and timing of this restructuring process in other mammals with different locomotor strategies and development. It is currently not clear whether there is a postnatal redistribution of cortical bone within the femoral diaphysis of chimpanzees as relative CA and medullary area (MA) metrics or cortical bone thickness have rarely been examined in ontogenetic samples and the sample size of chimpanzees under the age of 1 year is often very small (Bleuze 2024; Burgess 2018; Morimoto et al. 2018; Nadell, Elton, and Kovarovic 2021; Sarringhaus, MacLatchy, and Mitani 2016).

### 1.4 Cortical Bone Porosity

The resorption and deposition of cortical bone not only occurs at the inner and outer margins of the diaphysis but also internally within the cortex at the surfaces of vascular canals. Haversian canals, which house capillaries and nerves within each Haversian system or osteon, appear as pores in transverse diaphyseal sections. Cortical bone porosity, represented by the size, area, and number of these pores, as well as increasing trabecularisation at the endocortical boundary can change throughout life and can impact the structural integrity of bone (Ramchand and Seeman 2018). Clinical research investigating intracortical bone porosity in humans demonstrates a marked increase in porosity during skeletal aging because of increased bone resorption related to an uncoupling of osteoclastic and osteoblastic activity during bone remodeling (e.g., Zebaze et al. 2010). The mechanical implication of heightened porosity is an overall increase in bone fragility, as a combined result of a reduction to the load‐bearing area and increased susceptibility for crack propagation (Bala, Zebaze, and Seeman 2015; Yeni et al. 1997; Zimmermann, Busse, and Ritchie 2015). Additional research suggests that the amount of porosity is also sensitive to intense metabolic phases including accelerated growth periods or “growth spurts.” During these times of elevated calcium demands, it has been proposed that an increase in parathyroid hormone (PTH) stimulates the mobilization of calcium from bone tissue via increased intracortical bone resorption to enable rapid appositional and longitudinal growth (Parfitt 1994). Transient cortical bone porosity in humans has been demonstrated at both the infant growth spurt (Swan, Humphrey, and Ives 2023; Welsh and Brickley 2022) and pubertal growth spurt and has been linked to increased risk of bone fractures in adolescents (Kirmani et al. 2009; Parfitt 1994; Wang et al. 2010). To date it is not clear whether chimpanzees also undergo a similar pattern of skeletal growth. Previously, it has been established that chimpanzees increase in body mass around the time of puberty (Leigh 1996; Pusey et al. 2005) but whether there are phases of rapid increases in skeletal growth in length has been subject to debate (Hamada and Udono 2002; Watts and Gavan 1982). Longitudinal data on linear growth in non‐human primates is relatively rare and presents challenges when comparing between studies due to variation in methodology and analysis (See Berghaenel et al. 2023). An alternative approach for attempting to assess growth parameters by Sandel et al. (2023) uses urinary biomarkers of bone turnover, osteocalcin, and Type I collagen, to examine growth rates in a sample of 109 wild chimpanzees aged between 2.5–66 years over a duration of 2 years. In this study, a clear peak in osteocalcin and collagen was found at 9.4 and 10.8 years in male chimpanzees, coinciding with early and middle adolescence, but this pattern was not observed in females. Furthermore, the sample size for infants (0–5 years) was too small to accurately assess changes during early growth. It is therefore not fully understood whether a compensatory mechanism to facilitate rapid growth exists or is required in chimpanzees.

### 1.5 Aims and Objectives

Here, we aim to shed further light on the pattern of restructuring of cortical bone in the femur during the growth and locomotor development of the western chimpanzee subspecies, *Pan troglodytes verus* . This study will focus on a single subspecies, *P. t. verus*, to reduce the potential impact of interspecific variation on the results (Burgess 2018). As locomotor behavioral development has been previously documented in this subspecies in the wild (Bründl et al. 2021; Doran 1992, 1997), the western chimpanzee presents an opportunity to examine the timing of shifts in the structural arrangement of cortical bone and explore how these relate to the locomotor transitions documented in wild chimpanzees. Given that studies on primates have revealed differences in long bone CSG at different locations along the diaphysis (Nadell, Elton, and Kovarovic 2021; Swan et al. 2020), data will be collected from three separate diaphyseal locations to account for any regional differences in limb loading.

We aim to address the following questions:

1. Do changes in diaphyseal cross‐sectional shape of the femur in *P. t. verus* reflect the timing of locomotor transitions documented in wild chimpanzees?
2. Is there a postnatal increase in the relative contribution of medullary cavity to total subperiosteal area reflecting a shift in the cross‐sectional distribution of cortical bone? If so, do values rebound or stabilize as independent locomotion commences as has been documented in humans (Swan et al. 2020) and olive baboons (Cosnefroy et al. 2022)?
3. Are there any transient increases in cortical porosity within the studied age range and if so, do these reflect periods of increased metabolic activity associated with rapid skeletal growth?

## 2 Material and Methods

### 2.1 Sample

Data were collected from the femur of an ontogenetic series of 20 *P. t. verus* aged from newborn (2 weeks) to 12.6 years. Most of the *P. t. verus* sample (*N* = 18) derived from a single wild population from a long‐term study site in the Taï National Park, Republic of Côte d'Ivoire (Boesch, Wittig, and Crockford 2019). This population has been well researched since the late 1970s and data exists on age, sex, and cause of death for many of the individuals. Skeletons were recovered following deaths and are currently housed at the Max Planck Institute for Evolutionary Anthropology, Leipzig. This sample was supplemented with 2 wild‐shot *P. t. verus* individuals from Sierra Leone housed at the Natural History Museum, London. All selected femora were well‐preserved with no signs of pathology and were either unfused or not fully fused. Fully fused femora were considered adult and were not analyzed in this study. The left or right side was selected for study based on availability and preservation.

**FIGURE 2:**
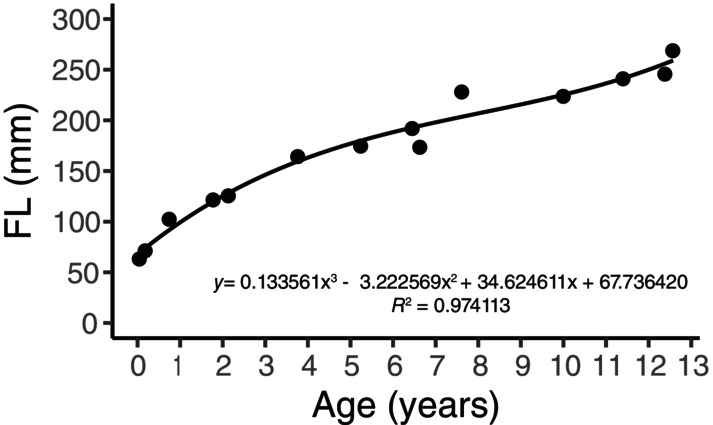
Femur length (FL) plotted against known chronological age. A third order polynomial curve was fitted to the data points and the equation was used to estimate age at death of unknown individuals.

Chronological age was determined for 14 individuals from the Côte d'Ivoire sample from documented dates of birth and death or dental histology (Neubauer et al. 2012; Smith and Boesch 2011). There is some variation in the precision of birth estimates. For some of the individuals birth date is known to the year only and month of birth is estimated (see Smith and Boesch 2011). For the remaining 4 individuals from Côte d'Ivoire and the 2 individuals from Sierra Leone, age was estimated based on femur length, measured as maximum intermetaphyseal length. Femur length was plotted against chronological age for the documented individuals and a third order polynomial curve was fitted to the datapoints (Figure 2). The equation of the curve was used to provide an estimate of age of the unknown individuals based on their femur lengths.

### 2.2 Locomotor Stages

Individuals were partitioned into locomotor stages based on age following previous observations of wild chimpanzees (Table 1). Stage 1 represents the first 5 months of life when the infant is unable to support its own body weight and locomote independently. During this time the infant is carried by its mother during transportation and stays in close proximity to the mother at all times (Doran 1992). Within Stage 1, infants demonstrate the emergence of postural skills including sitting up at an average age of 3.4 months and eventually standing up at an average age of 4.2 months (Bründl et al. 2021). Stage 2 (5 months—2.9 years) involves marked advances in both postural and motor skills including the emergence of climbing at an average of 5 months and first instances of independent walking (bipedal or quadrupedal) at an average of 6.7 months (Bründl et al. 2021). Stage 2 encompasses the onset of independent locomotor play and is defined by a diverse array of locomotor behaviors including climbing, torso‐orthograde suspensory, and quadrupedal and bipedal walking (Doran 1992; Sarringhaus, MacLatchy, and Mitani 2014; Van Lawick‐Goodall 1968). Of these behaviors, suspensory and climbing behaviors predominate their locomotor repertoire (Doran 1992; Sarringhaus, MacLatchy, and Mitani 2014). The beginning of Stage 3 (3–4.9 years) is characterized by a marked increase in terrestrial quadrupedalism, which comprises a considerably larger proportion of locomotor time than Stage 1 and 2 infants under the age of 3 years (Sarringhaus, MacLatchy, and Mitani 2014). Infants at this stage are still carried by the mother during travel (Doran 1992). Stage 4 (5–9.9 years) involves complete independence from the mother in terms of both locomotion and nutrition and this stage of life is frequently described as the juvenile period (Lonsdorf et al. 2020; Sandel et al. 2023; Sarringhaus, MacLatchy, and Mitani 2016). At this stage, quadrupedal walking predominates while progressively less time is spent on arboreal behaviors. Behavioral recordings of *P. t. schweinfurthii* subspecies suggest that this age group is characterized by a marked increase in quadrupedal running in comparison to those aged below 5 years (Sarringhaus, MacLatchy, and Mitani 2014). At Stage 5 (10–13 years), locomotor behavior patterns and frequencies closely resemble those of adults (Sarringhaus, MacLatchy, and Mitani 2014). The femur is not yet fully fused and chimpanzees in this age group have been considered as adolescents as individuals still tend to travel with their mothers or other caregivers (Sandel et al. 2023). On reaching adulthood, *P. t. verus* individuals will eventually spend approximately 86% of their time on quadrupedal behaviors (Doran 1996).

**TABLE 1:** Age categories representing locomotor stage reflecting changes in the type and frequency of mechanical loading of the femur.

Stage	Age range	N	Brief description of locomotor behaviors	References
1	0–4.9 months	2	Carried by mother during travel, stays close to mother. Engages in postural behaviors including sitting and standing up.	Bründl et al. (2021); Doran (1992)
2	5 months‐2.9 years	5	Takes first independent steps (quadrupedal and bipedal) and engages in a range of postural and locomotor behaviors including climbing, torso‐orthograde suspensory, and quadrupedal and bipedal walking.	Bründl et al. (2021); Doran (1992); Sarringhaus, MacLatchy, and Mitani (2014); Van Lawick‐Goodall (1968)
3	3–4.9 years	2	Transition toward increased quadrupedal walking and decreased suspensory behaviors. Still carried during travel.	Doran (1992); Sarringhaus, MacLatchy, and Mitani (2014)
4	5–9.9 years	7	Complete independence from mother. Increased frequency in quadrupedal walking and progressively less time spent on arboreal behaviors.	Doran (1997); Sarringhaus, MacLatchy, and Mitani (2014)
5	10–13 years	4	Close to adult locomotor behaviors. Mostly terrestrial quadrupedal walking.	Sarringhaus, MacLatchy, and Mitani (2014)

*Note:* Quadrupedal walking refers to both palmigrade and knuckle‐walking quadrupedalism.

### 2.3 CT Scan Processing

High resolution scans of the sample from the Taï Chimpanzee Collection were collected using a BIR ACTIS 225/300, diondo d3 and Skyscan 1173 high resolution micro‐CT scanner, at the Max Planck Institute for Evolutionary Anthropology (Leipzig, Germany). Scan parameters of 100‐140kv and 62‐140 μA with 810–3750 projections and a 1 mm aluminium, 0.5 mm brass or 0.25 mm brass filter were used. All scans were reconstructed as 8‐bit tiff stacks. The two Sierra Leone individuals (NHM_Z.D.1846.10.23.11 and NHM_Z.D.1976.436) were scanned at the Natural History Museum, London using a Nikon Metrology XT H 225 ST micro‐CT scanner. Scans were taken at 125 kV, 104 μA with 2000 projections and used a 0.5 mm copper filter. Full details of the scan resolution, sex, age, and locomotor stage for each individual are provided in Table 2.

**TABLE 2:** *P. t. verus* sample information.

ID	Sex	Age (years)	Side	Femur length (mm)	CT scan resolution (mm)	Locomotor stage
Taï Forest						
MPITC_11787	M	0.04^a^	L	63.1	0.015	1
MPITC_15015	—	0.18^a^	R	71.3	0.014	1
MPITC_15003	—	0.64^c^	R	88.6	0.016	2
MPITC_14993	F	0.74^a^	R	102.5	0.058	2
MPITC_15000	—	1.23^b^	R	105.6	0.058	2
MPITC_13432	M	1.77^a^	R	121.5	0.015	2
MPITC_11777	M	2.13^a^	R	125.4	0.015	2
MPITC_11788	F	3.76^a^	R	164.2	0.055	3
MPITC_14995	M	5.24^a^	L	174.7	0.091	4
MPITC_14992	—	5.84^c^	R	186.7	0.091	4
MPITC_11791	F	6.45^a^	L	192.1	0.091	4
MPITC_15011	M	6.63^b^	R	173.3	0.091	4
MPITC_13433	M	7.61^a^	L	228.0	0.091	4
MPITC_11782	M	8.34^b^	L	209.8	0.091	4
MPITC_15020	F	9.98^a^	L	223.5	0.091	4
MPITC_13437	F	11.4^a^	R	240.9	0.091	5
MPITC_11776	F	12.38^b^	R	245.4	0.091	5
MPITC_11779	M	12.57^b^	L	268.5	0.091	5
Sierra Leone						
NHM_Z.D.1846.10.23.11	—	3.99^c^	R	163.1	0.044	3
NHM_Z.D.1976.436	F	11.26^c^	R	239.7	0.065	5

^a^ Chronological age determined from birth and death dates or histological analysis (Smith and Boesch 2011).

^b^ Less accurate known age at death. Birth year known from year only with an error of ± 0.5 (Smith and Boesch 2011).

^c^ Age at death estimated from femoral length.

### 2.4 Data Processing and Analysis

**FIGURE 3:**
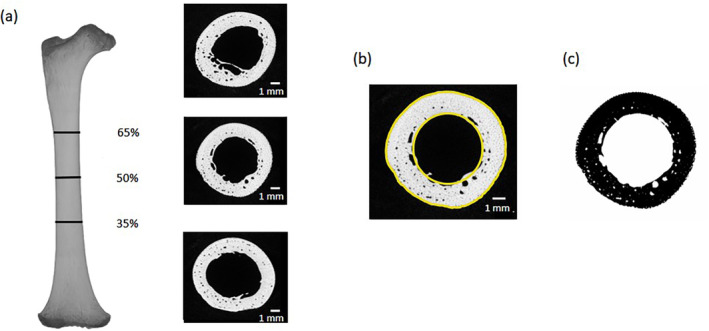
Example of the orientation of a juvenile chimpanzee femur from a CT scan and slice extractions from 35%, 50%, and 65% from distal end of diaphysis (a). Cross‐sectional parameters were quantified using a region of interest based on the subperiosteal border and an ellipse fitted internally to approximate the endocortical border (b). The image was binarized within the contours to estimate cortical bone porosity (c). Image displays MPITC 13432.

Micro‐CT scans of the femora were virtually orientated to a standardized position by initially aligning the bone by its principal axes using BoneJ v1 (Doube et al. 2010). Scans were then imported into Avizo Lite 9.2.0 (FEI Visualization Sciences Group), and each bone rotated about its long axis such that the most projecting points of the posterior intermetaphseal edge of the distal end of the bone were parallel to a straight line. For some older individuals, epiphyses were attached to the diaphysis due to connection via soft tissue or partial fusion. For these individuals, the epiphyses were removed by manual segmentation in Avizo as the presence of epiphyses would impact the consistency of the alignment procedure between individuals and length measurements. Maximum femoral length was defined as the maximum intermetaphyseal distance, which was measured as the length of the bounding box of the aligned bone. 2D slices were then extracted at 35%, 50%, and 65% of total intermetaphyseal length measured from the distal end (Figure 3a).

Cross‐sections were exported as TIFF files for cross‐sectional analysis in FIJI freeware (Schindelin et al. 2012). Each cross‐section was cropped, and the brightness and contrast levels were automatically optimized. The plugin, EPmacroJ (Sládek et al. 2018), was used to generate an external outline of the subperiosteal border and an ellipse was fitted internally to approximate the endocortical border (Figure 3b) using a minimum thresholding algorithm (Prewitt and Mendelsohn 1966). All recorded cross‐sectional parameters were quantified in EPmacroJ based on these outlines and included second moments of area (SMA) about the maximum (Imax) and minimum (Imin) axes, SMA about the mediolateral (Ix) and anteroposterior (Iy) axes, total area (TA), CA, MA, and cortical porosity area (CPA). CPA required a further processing step involving image binarization within the two outlines to separate bone material from air within the cortical boundaries (CA) (Figure 3c). Size‐independent ratios were used to examine ontogenetic changes in cortical bone structure. SMA ratios about the maximum and minimum axes (Imax/Imin) and mediolateral and anteroposterior axes (Ix/Iy) were used to provide an estimate of relative bending rigidity along the respective principal and anatomical axes and infer cross‐sectional shape. A medullary index, calculated as the ratio MA/TA, was used to estimate the relative contribution of the medullary cavity to the total subperiosteal area. This metric can be used to infer any relative expansion/contraction of the medullary cavity and in turn, the distribution of cortical bone from the neutral axis during appositional growth. A porosity index, calculated as CPA/CA, was used to estimate relative cortical bone porosity within the cortical area boundaries.

### 2.5 Data Analysis

Scatter plots of raw cross‐sectional area measurements (CA, TA, and MA) against age at death and femur length were generated and fitted with a local regression (LOESS) curve for each cross‐sectional location. Size‐independent cross‐sectional parameters (Imax/Imin, Ix/Iy, medullary index, and porosity index) were assessed using box‐and‐whisker plots for each locomotor stage. Statistically meaningful group comparisons could not be made due to small sample sizes within each subgroup, so ontogenetic patterns were visually assessed. Values of each variable were plotted against age at death to visualize the data independently of locomotor stage and LOESS curves were fitted. Graphical outputs were generated in R studio version 2023.06.1 (RStudio Team 2023).

## 3 Results

### 3.1 Cross‐Sectional Area

**FIGURE 4:**
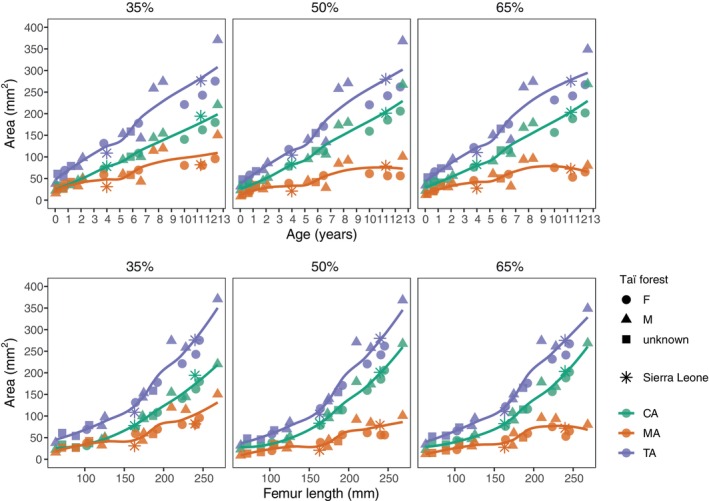
Scatter plots of total area, cortical area, and medullary area against age at death and femur length for each cross‐section location. LOESS curves were fitted to each variable using a smoothing parameter of 0.7. Individuals from the Taï forest are represented by filled datapoints with sex information indicated by shape. Individuals from Sierra Leone are represented by asterisks.

Scatter plots of the raw area measurements for CA, TA, and MA against age at death and femur length are displayed in Figure 4. All three area measurements demonstrate a consistent increase with age at all three cross‐sections with some sex‐based variability observed from about the age of 7 years involving a tendency for higher area measurements in males. All area measurements present a change in trajectory characterized by an increase in slope steepness between approximately 5 and 6 years, which is most marked in TA and MA. This trend is reflected and becomes more pronounced when plotted against femur length and occurs approximately between a growth attainment of 160–180 mm in femur length.

### 3.2 Relative Bending Rigidity

**FIGURE 5:**
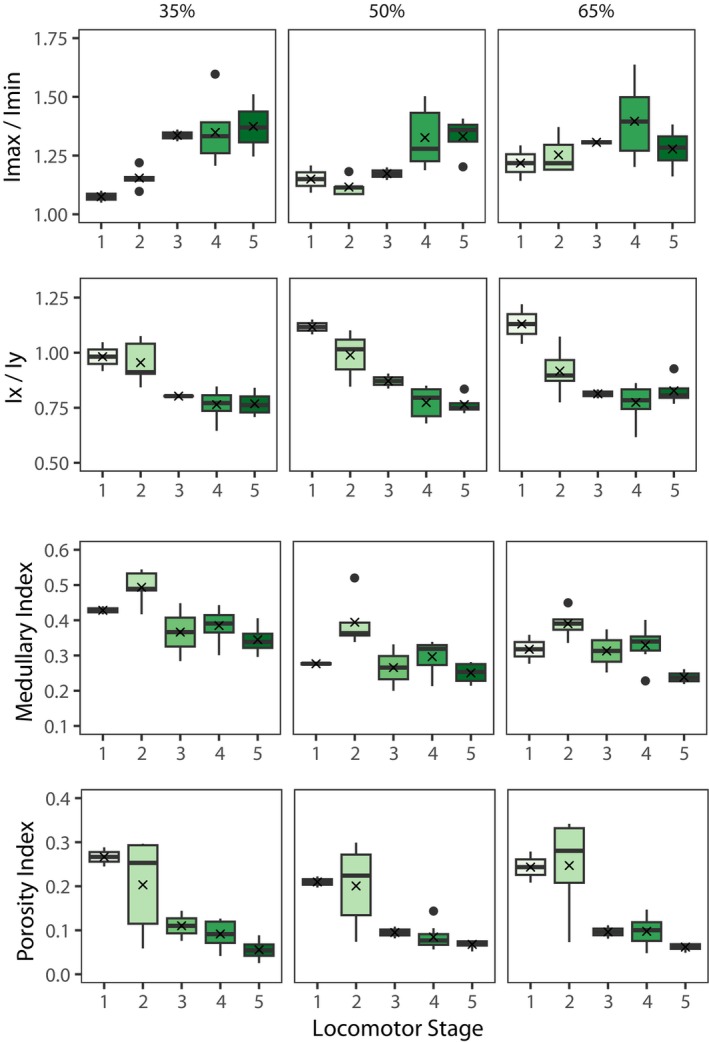
Box‐and‐whisker plots of Imax/Imin, Ix/Iy, medullary index, and porosity index against locomotor Stage 1 (0–4.9 months), Stage 2 (5 months—2.9 years), Stage 3 (3–4.9 years), Stage 4 (5–9.9 years), Stage 5 (10–13 years). The box represents the interquartile range and whiskers extends to values within 1.5× interquartile range from the upper and lower boundaries. Median and mean values are indicated within the interquartile range and are represented by a solid black line and a cross, respectively. Outliers include datapoints outside of the whiskers and are represented by black filled circles.

**FIGURE 6:**
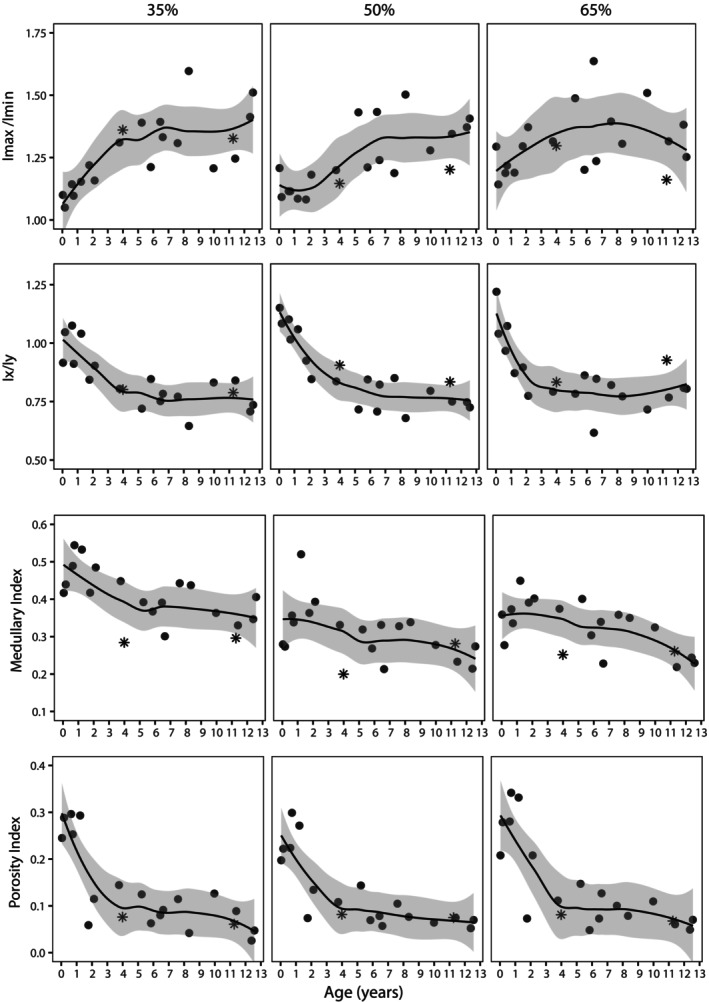
Scatter plots of Imax/Imin, Ix/Iy, medullary index, and porosity index against age at death. A Loess curves were fitted using a smoothing parameter of 0.7 and display a 0.95 confidence interval. Individuals from Sierra Leone are represented by asterisks.

Relative bending rigidity was assessed using SMA ratios Imax/Imin and Ix/Iy. Imax/Imin values are close to 1 in Stage 1 infants with the 35% exhibiting the most circular shape and 50% and 65% locations presenting slightly more elliptical (Figure 5). An increase in Imax/Imin is mostly demonstrated throughout the studied age range representing an increase in ellipticity at all cross‐section locations. Some deviations from this trend were observed including a small decrease in value from Stage 1 to Stage 2 at the 50% location and a decrease in value between Stage 4 and 5 at the 65% location. When considering SMA ratios about the anatomical axes (Ix/Iy), the results reveal an increase in mediolateral reinforcement along the length of the diaphysis. Scatter plots demonstrate that this increase in mediolateral eccentricity is most rapid during the first 5 years (Stages 1–3) but varies in pattern depending on cross‐section location (Figure 6). The 35% and 65% locations appear to stabilize between 2 and 4 years whereas a more gradual decline characterizes the midsection.

### 3.3 Relative Medullary Area

The medullary index, representing relative MA to total cross‐sectional area, presents dynamic changes within the studied age range at all three cross‐section locations (Figure 5). Average medullary index values show an increase between locomotor Stages 1 and 2 at all three locations, which is followed by a decrease between Stages 2 and 3. Median and average values remain lower than Stage 2 in subsequent stages but ranges are considerably overlapped. Scatter plots demonstrate a higher rate of decline during the first 5 years followed by a more gradual decline thereafter (Figure 6). The overall pattern suggests an expansion of the medullary cavity in the period from birth until the onset of independent locomotor behaviors in early infancy (Stage 2). The medullary index steadily decreases suggesting a contraction of the cavity as infants load their limbs, which continues at a lower rate as quadrupedal walking is refined and becomes the most dominant form of locomotion.

### 3.4 Intracortical Porosity

The porosity index, representing the relative area of porosity within the cortical cross‐sectional boundaries, was observed to vary across all five locomotor stages (Figure 5). Values generally declined across the stages of locomotor development for all cross‐section locations although a clear pattern is difficult to discern due to the large amount of variation in Stage 2. Ontogenetic patterns for porosity index are considerably clearer when the data are displayed as scatter plots (Figure 6). Individual data points reveal a high amount of cortical porosity from birth until approximately 1.5 years. Values rapidly decrease and stabilize at approximately 3–4 years at all cross‐section locations. From age 4 to 13 years, the values continue to decline but at a steadier rate.

## 4 Discussion

From birth through to adulthood, the growing chimpanzee skeleton must cope with the competing physiological demands of changing body mass, loading patterns, and hormonal conditions without compromising its overall structural integrity. The results from this study indicate a series of structural phases in cortical bone development for all studied parameters, which collectively suggests a degree of anatomical integration that optimizes bone tissue distribution during periods of rapid growth and supports rapid changes in locomotor development.

### 4.1 Cross‐Sectional Area

While cross‐sectional area measurements increase with age and increasing bone length throughout growth, the data presented here suggest differences in the rate of growth in TA and MA, which appears to accelerate from approximately 5 and 6 years of age. This pattern is most clearly visible when area measurements are plotted against femur length (Figure 4). Interestingly, this accelerated phase of development broadly coincides with the time of complete independence from the mother in terms of both locomotion (i.e., carrying) and nutrition (Doran 1992; Nishida et al. 2003; Sarringhaus, MacLatchy, and Mitani 2014). It is possible that this shift is influenced by a higher frequency of repeated quadrupedal loading associated with a higher peak vertical force on the hindlimb in addition to changes in body mass and that collectively these factors contribute to a greater consolidation of cortical bone structure in juveniles.

### 4.2 Relative Bending Rigidity

In terms of relative bending rigidity, both SMA ratios (Imax/Imin, Ix/Iy) reveal changes throughout growth, indicating an increase in asymmetry along the mediolateral plane during early ontogeny at all three cross‐section locations, which is consistent with previously documented trends (Bleuze 2024; Burgess 2018; Morimoto et al. 2018; Sarringhaus, MacLatchy, and Mitani 2016). In terms of variation in shape along the diaphysis, these results differ to the findings by Nadell, Elton, and Kovarovic (2021), in that the distal location did not remain static during growth and instead reflected similar developmental shifts to the mid‐shaft and proximal locations. Additionally, the present findings suggest a more circular cross‐sectional shape in the distal location of the youngest age category (0–4.9 months) than 50% and 65% locations that were slightly more AP reinforced. This contrasts to the more ML reinforced cross‐section at the distal location in the youngest age group (0–5 years) in Nadell, Elton, and Kovarovic (2021). When considering these differences, it is important to acknowledge that the distal location examined here was extracted more proximally, at 35% of diaphyseal length, compared to Nadell, Elton, and Kovarovic (2021), where it was taken closer to the metaphysis at 20% of diaphyseal length. The 20% distal location, which was not included in this study, may be subject to greater anatomical constraint due to closer proximity to the knee joint (Nadell and Shaw 2016). It is also possible that the smaller age group divisions used in this study revealed more subtle changes in diaphyseal shape during early development that could not be detected within a broader 5‐year age group.

The pattern and timing of changes in relative bending rigidity documented here do appear to correspond with changes in locomotor development. Cross‐sectional shape is near circular in the first two stages during which the femur is subjected to minimal loading and variable mixed loading during early independent locomotor behaviors. Diaphyseal shape becomes increasingly more elliptical as a likely reflection of less variable loading patterns of the hindlimbs as terrestrial quadrupedal walking increases and arboreal behaviors become less frequent. The Imax/Imin and Ix/Iy scatter plots in Figure 6 suggest that shape changes toward ellipticity are most prominent during the first 5 years and is followed by a stabilization of values. Although there is some variation on timings depending on cross‐section location, the results suggest an attainment of an adult‐like diaphyseal shape prior to skeletal maturation, which is consistent with previous datasets that have documented no statistical differences in mean femoral midshaft shape values between juveniles (5–10 years) and adults (~15 + years) (Bleuze 2024; Sarringhaus, MacLatchy, and Mitani 2016).

The results here are of interest as there has been prior debate regarding whether an increase in mediolateral eccentricity of the femur is related to locomotor changes, changes in body mass or is a product of genetic control. Morimoto and colleagues suggested that femoral diaphyseal shape is primarily genetically determined as distinct ontogenetic trajectories have been observed in chimpanzees and gorillas despite similarities in adult locomotor behaviors (Morimoto et al. 2018). Yet, as the pattern and timing of changes in cross‐sectional shape appear to follow locomotor transitions documented in the wild (Doran 1992; Sarringhaus, MacLatchy, and Mitani 2014), the results from this study suggest that diaphyseal shape is, to some degree, influenced by mechanical loading. While rapid changes in body mass likely contribute to femoral diaphyseal form during growth (Nadell, Elton, and Kovarovic 2021; Ruff and Runestad 1992), the notable increase in mediolateral reinforcement observed here is mostly confined to the first 5 years when quadrupedal behavior progressively increases in frequency and is shown to stabilize prior to the increase in appositional growth velocity represented by total subperiosteal area (Figure 4). As highlighted recently by Bleuze (2024), it is important to bear in mind that there are some discrepancies among studies in how well cross‐sectional properties track locomotor transitions during chimpanzee growth (Bleuze 2024; Morimoto et al. 2018; Nadell, Elton, and Kovarovic 2021; Sarringhaus, MacLatchy, and Mitani 2016). Any functional interpretation should be made with caution as the combined influence of multiple factors on diaphyseal shape is currently not well understood and, ultimately, data on kinematic information on joint loading during chimpanzee development is lacking.

### 4.3 Relative Medullary Area

Previous research on humans has demonstrated an enlargement of the medullary cavity and redistribution of cortical bone from the endosteal to subperiosteal margins between birth and 6 months (Swan et al. 2020). The results of the current study suggest that chimpanzees may also undergo a similar pattern of restructuring during early growth. Compared to Stage 2 infants, the proportion of MA to CA of chimpanzees is relatively small in individuals under 5 months, which suggests a thick band of cortical bone surrounding a relatively narrow medullary cavity is present at birth. Research on humans, suggests an in utero increase in osteoblastic activity at the endocortical margin due to exposure to placental estrogen (Rauch and Schoenau 2001) leading to what has been referred to as a phase of “gestational overproduction” of bone (Acquaah et al. 2015). The removal of placental estrogen and shift to an environment with low mechanical resistance following birth is accompanied by increased resorption of bone at the endocortical margin, which alters the spatial distribution of cortical bone tissue (Rauch and Schoenau 2001). This offers a mechanical advantage to the developing skeleton by creating a structure capable of resisting loads efficiently with minimal bone mass as cortical bone is displaced further from neutral axis (Kontulainen et al. 2007; Rauch and Schoenau 2001). A large amount of cortical bone at birth provides a calcium reservoir to support mineral homeostasis, which is particularly beneficial during this life stage due to the high metabolic demands associated with rapid growth (Rauch and Schoenau 2001). As infants begin to mechanically load their limbs, the relative amount of MA to CA stabilizes or begins to decrease.

The pattern of changing cortical bone distribution during infancy recorded within this ontogenetic sample of *P. t. verus* appears to echo that previously observed in humans (Swan et al. 2020). Here, average medullary index presents an increase between Stages 1 and 2 at all three cross‐section locations but is particularly marked at the 50% location. A peak is reached at Stage 2 followed by a steady decline between Stages 2 and 3 coinciding with the onset of independent locomotion and engagement with arboreal and suspensory behaviors. It is important to bear in mind that the sample size is particularly low in the youngest age groups with Stage 1 and Stage 3 infants each represented by just two individuals. An estimation of age at the point of inflection could not confidently be made but the results tentatively suggest that a restructuring of cortical bone occurs within the first 3 years.

Given the altricial nature of locomotor development for both chimpanzees and humans, it is perhaps not surprising that both species could share a pattern of bone restructuring that allows phenotypic plasticity and optimisation of bone mass during changing loading conditions. Yet this patterning does not appear to be consistent across all regions of the femur. In contrast to humans, trabecular bone in the proximal femur of chimpanzees does not appear to reduce in bone volume fraction in early infancy (Tsegai et al. 2018a). This led Tsegai et al. (2018a) to suggest that all taxa could potentially undergo such a phase of bone volume reduction, but this may occur at different developmental stages, occurring post‐birth in humans, for example, but in utero in chimpanzees.

While possible species‐related differences may contribute to the discrepancy between cortical and trabecular bone restructuring patterns, there may be regional differences within an element that could complicate ontogenetic trends. Previous research has assessed the extent of regional variation in trabecular bone across different skeletal regions (Chirchir 2019; Tsegai et al. 2018b) and within the femoral neck. In the latter, Milovanovic et al. (2017) found decreases in bone volume fraction in humans during the first 6 months in most regions of the femoral neck except for one region directly adjacent to the epiphyseal plate in the proximal femur. This region showed high trabecular bone number and low trabecular bone separation, and these authors suggested this structure represented the dominance of new bone formation during endochondral bone development as adjacent to the growth plate (see also Ryan and Krovitz 2006). It is therefore feasible that some variation in ontogenetic patterns could exist depending on the positioning of regions of interest and the underlying bone structures being captured during development.

It is also worth noting the functional, locational, and morphological differences between cortical and trabecular bone types that may influence differences in bone restructuring. Cortical bone along the diaphysis is primarily adapted to resist bending loads and some compression, whereas the trabecular bone functions to provide strength and distribute external loads away from the joint (Kivell 2016). These two bone types may not always covary in response to loading history due to differences in the rate of remodeling and variation in loading experienced at the more dense cortical diaphysis in comparison to the cancellous rich articular sites (Arlot et al. 1997; Murray and Erlandson 2022; Shaw and Ryan 2012). Further work is required to assess patterns of variation more fully to explore differences between trabecular and cortical bone development in the chimpanzee femur and studies would benefit from examining variation in bone structure at multiple long bone sites across different species to examine why such patterns exist.

### 4.4 Intracortical Porosity

**FIGURE 7:**
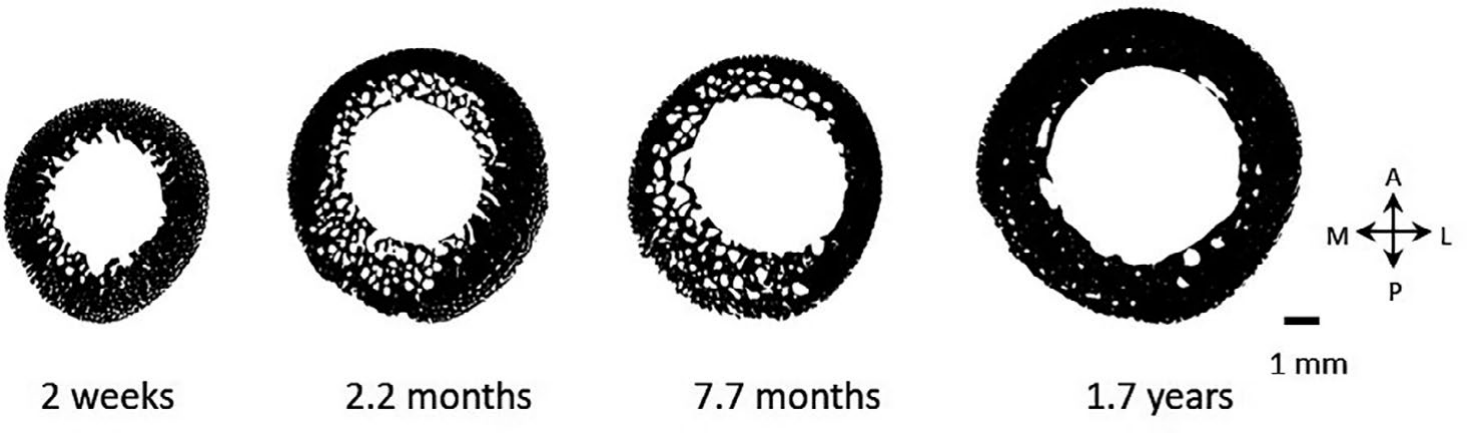
Examples demonstrating changing intracortical porosity in chimpanzees during the first 2 years at the femoral midsection. Individuals include MPITC_11787, MPITC 15015, MPITC 15003, MPITC 13432. Cross‐sections are right orientated and anatomical directions are indicated (A, anterior; M, medial; P, posterior; L, lateral).

Similar to relative MA, intracortical porosity was also characterized by dynamic changes during early growth. Porosity index values are highest during the first 1.5 years of life followed by a rapid decline, which stabilizes at approximately 3–4 years coinciding with the marked increase in time spent on quadrupedal behaviors (Sarringhaus, MacLatchy, and Mitani 2014). As there are only two individuals representing Stage 1 (0–5 months), it is difficult to conclude whether high porosity is present at birth or whether there is an increase during the first few months followed by a decline. Although the sample size was not large enough to pinpoint more precisely the timing at which porosity levels decline, the data suggests a transient period of elevated porosity during early postnatal growth (Figures 6 and 7). As has been observed in humans, an increase in cortical bone resorption coinciding with rapid growth during infancy likely acts to help maintain mineral homeostasis but at the expense of forming larger canals within the cortex and coalescing canals at the endocortical boundary (Swan, Humphrey, and Ives 2023; Welsh and Brickley 2022). The presence of this pattern of changing cortical porosity suggests chimpanzees also possess a compensatory mechanism to facilitate rapid growth during infancy. Whether this confers a significant structural disadvantage in terms of skeletal fragility and fracture susceptibly at this age group is not known. Infants aged from 5 months to 2.9 years are still partly dependent on adults for locomotion and have anecdotally been observed to move cautiously through the trees when feeding under the guidance of the mother (Van Lawick‐Goodall 1968). Cautious behavior in addition to small body weight may engender a lower risk of locomotor‐related injuries such as falls related to weak branches.

It is also important to bear in mind that, as well as structural properties, the material properties of bone vary throughout life and may also impact skeletal resistance to fracture (Currey 2003). In humans, it has been demonstrated that immature bone is generally more compliant and tougher than mature bone and has a greater capability to absorb higher energy loads without failure because it is relatively less mineralized (Currey 1979, 2003). In contrast, the bone of precocial animals such as deer, is more mineralized in neonates and has a higher Young's modulus, which helps withstand high mechanical forces and maximize locomotor efficiency soon after birth (Currey and Pond 1989; Heinrich, Ruff, and Adamczewski 1999; Wei and Zhang 2019). It is possible that the material properties of bone tissue at birth in some altricial species could be a mechanism for adaptation to changing locomotor behaviors, as less stiff bone with an ability to absorb higher energy may be beneficial for resisting fractures related to the potential tumbles and falls associated with learning to walk (Currey 1979). Furthermore, a high porosity in young bone does not, in a material sense, impact energy absorption (Currey 1979). It is therefore unlikely that the high porosity demonstrated in young chimpanzees would have a negative impact on the structural integrity of bone at this life stage. Potential changes in the material properties of cortical bone during ontogeny in chimpanzees are not well studied and the impact of these and variation in porosity on the mechanical properties of bone warrants further exploration.

Interestingly, in this sample there was no evidence to suggest a secondary transient increase in cortical porosity occurring in pubescent chimpanzees as has been shown in humans (Kirmani et al. 2009; Parfitt 1994; Wang et al. 2010). There is a female bias (3/4) in the relevant age group (age 10–13 years) and the results are consistent with previous research that suggests an absence of increased bone turnover in pubescent females (Sandel et al. 2023). Although Sandel et al. (2023) identified a clear peak in urinary markers indicative of bone turnover in adolescent male chimpanzees, they did not find a conclusive pattern in females. This concords with research on ontogenetic changes in body mass in captive common chimpanzees where a clear peak in growth velocity is present in males at around 10 years of age but is absent in females (Leigh 1996). Unfortunately, any potential pubertal shift in bone porosity in males is likely to be masked in this study sample. It is also possible that the cross‐sectional nature of this study and that of previous studies such as Leigh (1996) has a minimizing effect on the perceived magnitude of a potential growth spurt because of variation in the timing of puberty between individuals, as has been suggested in adolescent humans (Tanner 1986). This risk is exacerbated by the use of rather broad age groups, which are necessary with small sample sizes. Further research would benefit from an increased sample size of prepubescent and pubescent individuals and a distinction between males and females to shed light on the potential for sex‐based differences in chimpanzee growth pattern and velocity.

### 4.5 Limitations

The results from this study provide further insights into cortical bone structural development in wild chimpanzees and the inclusion of multiple age categories during the first 5 years reveals a nuanced perspective on the early phases of growth. There are, however, several limitations relating to both the sample and methodology which should be considered when interpreting the findings of this study. It is first worth noting that while the use of micro‐CT scans provides a non‐destructive means of examining internal bone parameters, the accuracy of quantifying very small features such as cortical bone pores can be restricted by scan resolution, which varies across the sample. Furthermore, the use of an automated ellipse to define the endocortical boundary may also introduce a degree of error when calculating medullary and porosity area, and SMA measurements. While this technique enables a quick and objective method of recording, it may oversimplify the border and may not accurately capture the outline of zones of increased trabecularization in bone, which can develop at the endocortical margin (see Swan, Humphrey, and Ives 2023).

An additional limitation of this research is the small sample size. There are relatively few nonadult postcranial skeletons of primates in museum and university collections, and the Taï sample is the largest collection of chimpanzees (to our knowledge) of known age and sex. For this study, the sample was deliberately not combined with individuals that lived in a captive environment as studies suggest marked differences in the growth and fusion rates of the skeleton, with captive chimpanzees typically presenting more precocious skeletal development (Zihlman, Bolter, and Boesch 2007). Research also suggests a potential for differences in bone microstructure between some wild and captive mammalian species due to variation in vivo forces and locomotor ecology (Zack, Smith, and Angielczyk 2022). In addition, as differences in some parameters of long bone cross‐sectional geometry have been demonstrated between different subspecies of wild adult and juvenile chimpanzees due to potential differences relating to ecology, time spent on arboreal behaviors, overall activity, and underlying genetic influences (Burgess 2018; Carlson et al. 2011), no attempt was made to combine subspecies. As a result of small sample size there is a low and uneven representation of individuals across the studied age range, and this may limit understanding of the extent of variation within the subspecies *P. t. verus* during growth. Observed trends, particularly during infancy, should be treated with caution considering that Stage 1 infants are represented by just two individuals aged 2 weeks and 2 months and Stage 3 infants are represented by two individuals aged 3.8 years and 4 years.

Similar to other ontogenetic studies on chimpanzee cortical bone structure (Bleuze 2024; Sarringhaus, MacLatchy, and Mitani 2016), this study has utilized age categories defined by stages of locomotor development to provide an analytical framework. Data are mostly derived from Doran (1992) and Sarringhaus, MacLatchy, and Mitani (2014), which have provided invaluable insights into chimpanzee locomotor development. Behavioral observations are mostly in agreement although it is important to acknowledge that there is a discrepancy regarding the transition to increased terrestrial quadrupedalism during infancy with Doran (1992) suggesting a sharp increase from 2 years and Sarringhaus, MacLatchy, and Mitani (2014) suggesting a more gradual increase from 3 years. The increased sample size adopted in Sarringhaus, MacLatchy, and Mitani (2014) is likely to be less impacted by individual variation, but it is unclear whether there is variation between sites and subspecies. The impact of *Pan* subspecies variation on cross‐sectional geometry during ontogeny has started to be explored by Burgess (2018). Future studies on bone functionality during growth would benefit from further comparative behavioral observations.

Lastly, a degree of variation and a small number of extreme outliers were identified in the dataset. There are several factors that may contribute to this variation. Causes of death varied across the sample and included both unnatural causes related to hunting but also natural causes such as infectious disease and starvation (see Supporting Information in Tsegai et al. 2018a). The inclusion of individuals that died from natural causes means that there is an unavoidable degree of mortality bias. We do not know the extent of potential underlying health issues on bone modeling and remodeling, but this should be acknowledged when interpreting the results as has previously been discussed in the context of human archeological samples (e.g., Humphrey 2000; Wood et al. 1992). Although most of the sample included *P. t. verus* individuals from a single region (Taï forest in Côte d'Ivoire), it is possible that a degree of variation in cortical bone structure may exist between this group and the individuals from Sierra Leone due to environmental differences between site locations, such as terrain and the amount of tree coverage, which may impact mechanical loading. Furthermore, while chronological age was known for the majority of the sample, age estimates from femoral length from the remainder of the sample may contribute to some variation in the dataset due to the sensitivity of this parameter to environmental stressors (Cardoso 2007; Humphrey 2000). The continuation and support of long‐term field sites and ongoing retrieval of primate remains subsequent to known deaths would prove fruitful for understanding the impact of these factors on bone (re)modeling and future research on locomotor and skeletal development.

## 5 Conclusions

Cortical bone structure represented by bending rigidity, relative MA, and cortical bone porosity were assessed in an ontogenetic sample of a single chimpanzee subspecies (*P. t. verus*). The results demonstrate rapid changes in cortical bone structure during the first few years of life involving dynamic changes in relative MA and cortical bone porosity during the time that chimpanzees engage in variable, but independent arboreal and suspensory behaviors and are occasionally carried by adults. Diaphyseal cross‐sectional shape demonstrates an increase in mediolateral reinforcement during the first 5 years followed by a stabilization once quadrupedal walking is well established as the dominant locomotor mode (Sarringhaus, MacLatchy, and Mitani 2014). The results illustrate a degree of developmental plasticity of the femur that enables a response to changing hormonal, mechanical, and metabolic influences associated with rapid growth without compromising long bone functionality. This research also highlights the importance of accounting for rapid changes in cortical bone structure during the earlier phases of chimpanzee growth, which is particularly relevant for the selection of age categories during the first 5 years. The structural changes recorded here and those identified previously in modern humans present both commonalities and differences, which are not yet fully understood. The varying degree and complex interaction of both mechanical and nonmechanical factors should be considered with caution in any application of these findings in the reconstruction of locomotor behavior and skeletal development in fossil hominins.

## Author Contributions

**Karen R. Swan:** conceptualization (equal), data curation (equal), formal analysis (equal), investigation (equal), methodology (lead), writing – original draft (lead), writing – review and editing (equal). **Zewdi J. Tsegai:** conceptualization (equal), data curation (equal), investigation (equal), methodology (supporting), writing – review and editing (equal). **Rachel Ives:** conceptualization (supporting), investigation (equal), writing – review and editing (equal). **Louise T. Humphrey:** conceptualization (equal), formal analysis (equal), funding acquisition (lead), investigation (equal), writing – review and editing (equal).

## Ethics Statement

The authors have nothing to report.

## Conflicts of Interest

The authors declare no conflicts of interest.

## Data Availability

The data that support the findings of this study are available from the corresponding author upon reasonable request.
